# RETRACTION: Integrated Strategies for the Clinical Correlation, Prevention and Management of Chronic Oral Infections, Wounds and Arteriosclerotic Occlusion in Lower Extremities

**DOI:** 10.1111/iwj.70491

**Published:** 2025-04-02

## Abstract

**RETRACTION:** LinF. , HanJ. , XueH. , KeJ. , ZhangX. , and LinM. , “Integrated Strategies for the Clinical Correlation, Prevention and Management of Chronic Oral Infections, Wounds and Arteriosclerotic Occlusion in Lower Extremities,” International Wound Journal 21, no. 2 (2024): e14669, 10.1111/iwj.14669.PMC1082756442052959

The above article, published online on 30 January 2024, in Wiley Online Library (http://onlinelibrary.wiley.com/), has been retracted by agreement between the journal Editor in Chief, Professor Keith Harding; and John Wiley & Sons Ltd. Following an investigation by the publisher, all parties have concluded that this article was accepted solely on the basis of a compromised peer review process. The editors have therefore decided to retract the article. The authors did not respond to our notice regarding the retraction.

**RETRACTION:** F. Lin , J. Han , H. Xue , J. Ke , X. Zhang , and M. Lin , “Integrated Strategies for the Clinical Correlation, Prevention and Management of Chronic Oral Infections, Wounds and Arteriosclerotic Occlusion in Lower Extremities,” International Wound Journal 21, no. 2 (2024): e14669, 10.1111/iwj.14669.PMC1082756442052959

The above article, published online on 30 January 2024, in Wiley Online Library (http://onlinelibrary.wiley.com/), has been retracted by agreement between the journal Editor in Chief, Professor Keith Harding; and John Wiley & Sons Ltd. Following an investigation by the publisher, all parties have concluded that this article was accepted solely on the basis of a compromised peer review process. The editors have therefore decided to retract the article. The authors did not respond to our notice regarding the retraction.

